# Extracts from Our Correspondents

**Published:** 1872-03

**Authors:** 


### EXTRACTS FROM OUR CORRESPONDENTS.

---

Dr. E. T. McSwain, of South Carolina, says:

"I have been most heartily pleased with The Companion. Its printing is good, its matter is excellent, and the doctrines inculcated are such as are for the good of the profession and humanity.

"I have been all my life a reader of the medical journals of the country, but none I have ever seen heretofore, seem to me so well adapted to the busy practitioner as The Companion. Its articles are short and practical, its formulæ and prescriptions all from the best authority, and of the greatest value. And right here occurs a thought: A most serious difficulty under which all practitioners in the South have had to labor, is that all, or nearly all, the books, journals, and other literature of the profession, have been compiled and written in other lands, and we have had to adopt our own theory and practice to a large extent in the South; but The Companion is contributed to largely by Southern practitioners, who are familiar with Southern diseases, and who give the treatment that is successful in a Southern climate. This I consider a most important matter; for we all know that diseases differ in different climates, and a disposition on the part of Southern practitioners to be content with their own success, and not trouble to publish it to the world, has kept the profession in ignorance, to a large extent, of the many affections peculiar to us; and many a physician who has been most fully conversant with the etiology and treatment of many diseases of a local character in the South, has not been known fifty miles from home. But I trust that the day is past, and that through The Companion, and, if necessary, through other channels, we may make a medical literature of the South that will be replete with information to all of us, and do honor to our much loved South.

In a future article I will furnish you somewhat with the climate, diseases and their treatment of this section."

"I fully endorse the statement made in your February number, viz: 'No institution can succeed unless it be conducted upon principle of right and justice'; and I think it is high time that a programme of rules and principles were adopted by which all regular recognized colleges should be controlled and governed. It is true the States have a right to charter any number of colleges of all systems, and to give their trustees or faculties such powers and privileges as the Legislature "may see fit and proper," but it is the right and privilege of the profession to say upon what conditions a college and its students may be recognized.

"1st, Its charter should be in harmony with the laws or ethics of the profession. It should neither give to faculty or trustees the power by which they could torture those principles common to Christianity and modern civilization.

"2nd, The professors should be men of medical learning and the highest order of morals—"known and read of" all men as unyielding supporters of the "golden rule" of ethics—medical and Christian.

"3d, The trustees should be the warm and honest guardians of the interests of the profession, and their entire aim should be to aid the faculty in carrying out the most exalted views of the profession, of whose honor and dignity they are the authorized protectors. They should be men of high, noble views, impelled by hearts above petty influences, and alive to the reputation of the great charge committed to their trust. The character of an institution can be as effectually ruined and destroyed by a narrow-minded, selfish board of trustees, as by an undeserving faculty; and when both are combined, its doom may be predicted as near at hand."

We endorse the views of our correspondent, and have no doubt the profession will say, Amen. We have another letter upon this subject, which, for want of space, cannot appear in this number. We will give our views upon the subject in our next.

